# Anterior Midline Skull Base Meningiomas: A Systematic Review of Resection Rates, Functional Outcomes, and Perioperative Complications Following Contemporary Endoscopic Endonasal Versus Transcranial Approaches

**DOI:** 10.3390/jcm15124676

**Published:** 2026-06-16

**Authors:** Umid Sulaimanov, Irem Uslu, Omar Alomari, Yerkebulan Serikkanov, Mariagrazia Nizzola, Darius Ansari, Oyku Ozturk, Nafiye Sanlier, Ahmed Rasim Bayramoglu, Eray Tekirdas, Abdullah Keles, Ufuk Erginoglu, Mustafa K. Baskaya

**Affiliations:** 1 Department of Neurological Surgery, School of Medicine and Public Health, University of Wisconsin, Madison, WI 53792, USAserikkanov@wisc.edu (Y.S.);; 2 Hamidiye International School of Medicine, University of Health Sciences, Istanbul 34668, Türkiye

**Keywords:** anterior skull base meningioma, olfactory groove meningioma, tuberculum sellae meningioma, planum sphenoidale meningioma, endoscopic endonasal approach, transcranial approach, nasoseptal flap, cerebrospinal fluid leak

## Abstract

**Objectives**: Anterior midline skull base meningiomas, such as olfactory groove meningiomas (OGMs), planum sphenoidale meningiomas, and tuberculum sellae meningiomas pose significant surgical challenges due to their proximity to neurovascular and olfactory structures. The widespread use of the vascularized nasoseptal flap (NSF) has expanded the role of the endoscopic endonasal approach (EEA). This systematic review compares outcomes of EEA and the transcranial approach (TCA) in the NSF era. **Methods**: PubMed, Embase, and Scopus were searched following PRISMA guidelines for studies published January 2010 and August 2025 comparing EEA and TCA for OGMs and tuberculum sellae/planum sphenoidale (TS/PS) meningiomas with NSF reconstruction. Thirty-five studies (1866 patients) were included and outcomes were stratified by tumor subtype. **Results**: Gross total resection (GTR) exceeded 90% across both approaches. EEA was associated with superior visual improvement in TS/PS meningiomas, particularly with optic canal involvement, while TCA resulted in better olfactory preservation in OGMs. Cerebrospinal fluid (CSF) leak rates have declined substantially with vascularized NSF reconstruction, especially for TS/PS meningiomas. **Conclusions**: Both EEA and TCA achieve high GTR rates for anterior midline skull base meningiomas. EEA provides advantages for visual outcomes in TS/PS meningiomas, whereas TCA are favored for olfactory preservation in OGMs. Approach selection should be individualized based on tumor anatomy and functional priorities.

## 1. Introduction

Anterior midline skull base meningiomas, including olfactory groove meningiomas (OGMs), planum sphenoidale meningiomas (PSMs), and tuberculum sellae meningiomas (TSMs), constitute a distinct subset of anterior cranial base tumors with complex anatomical relationships [1]. Owing to their relatively silent early disease course, patients with OGMs and PSMs frequently present with very large tumors and may be associated with hyperostotic changes in the skull base [2,3,4]. In contrast, TSMs may become symptomatic at a smaller size due to their proximity to the optic chiasm, with visual dysfunction often manifesting early in the disease course. Regardless of size, the unique location of these meningiomas in the anterior cranial fossa often compromises critical neurovascular structures, such as the optic apparatus, anterior cerebral arteries (ACAs), and olfactory tracts, among others [5]. Paramount to their surgical management, then, is to alleviate pathologic compression and minimize risk of recurrence through maximal tumor resection, all while preserving neurological and vascular function [1,6].

Transcranial approaches (TCAs), such as subfrontal, pterional, and bifrontal craniotomies, offer wide exposure and facilitate early vascular control, lateral access, and aggressive bony decompression [1]. Endoscopic endonasal approaches (EEAs), including extended variants, have emerged as “minimally invasive” alternatives owing to their direct midline trajectory, early decompression of the optic canal, and avoidance of brain retraction [7,8]. However, the principal limitation of EEAs has historically been a high risk of postoperative cerebrospinal fluid (CSF) leakage. The introduction of the vascularized nasoseptal flap (NSF) has engendered increasing adoption of EEAs for skull base reconstruction, driven largely by reduced rates of postoperative CSF leak [9]. Subsequently, the advent of these techniques has led to expanding indications of EEAs to include anterior midline skull base meningiomas that were traditionally considered suitable solely for open TCAs.

Nevertheless, reconstruction techniques remain heterogeneous across institutions: variations in flap design, multilayer closure, and adjunctive measures continue to influence outcomes and reproducibility, even within the NSF era.

Despite these technical advancements, the optimal surgical corridor for anterior skull base meningiomas remains controversial. Although improvements in NSF techniques have facilitated the more widespread indications of EEAs, it is critical that the choice of approach is guided by patient- and tumor-specific features such as size, lateral extension, skull base involvement, and feasibility of reconstruction, and functional goals. As an example, approaches to OGMs may be driven by considerations such as olfactory preservation [10].

To this end, we conducted a contemporary systematic review of the anterior midline skull base literature, focusing on comparative outcomes of EEAs and TCAs with regard to extent of resection, neurological outcome and tumor recurrence, complication rates, and CSF leak. Rather than identifying a universally superior approach, our objective was to evaluate how the adoption of the NSF has reshaped these operative and functional outcomes. Where possible, we stratified data by tumor subtype, specifically OGMs, TS/PS meningiomas, to reflect their distinct surgical and functional challenges. Importantly, tuberculum sellae meningiomas (TSMs) and planum sphenoidale meningiomas (PSMs) represent anatomically and clinically distinct entities. TSMs originate at the junction of the chiasmatic sulcus and tuberculum sellae and are uniquely positioned to compress the optic chiasm and invade the optic canals, making visual dysfunction the hallmark presenting symptom. PSMs arise more anteriorly along the planum and more commonly produce cognitive, behavioral, or frontal lobe symptoms, with visual involvement occurring less frequently and typically at a later stage. Despite these differences, the two subtypes share a common surgical corridor and are often pooled in the literature due to the relatively low frequency of isolated PSM series [6,11,12]. Where data permitted, this review attempted subgroup stratification; however, the majority of included studies reported outcomes for TSMs and PSMs together, necessitating their joint analysis in most comparisons [1,6,11].

Despite these advancements, several specific controversies continue to motivate further investigation. First, whether the vascularized nasoseptal flap has sufficiently mitigated the historical CSF leak risk of EEAs to justify its expanded indications remains debated, given that residual leak rates of approximately 10% persist in many contemporary cohorts [1,13]. Second, the visual outcome advantage frequently attributed to EEAs in TS/PS meningiomas may, at least in part, reflect the inconsistent application of early optic canal decompression in transcranial series, which has been shown to independently predict visual improvement [14,15,16]. Third, whether meaningful olfactory preservation is achievable through EEAs in OGMs remains contested, with anosmia rates approaching 100% in most endonasal series compared with substantially better preservation following unilateral transcranial approaches [10,17]. Fourth, although feature-based selection algorithms have been proposed [12], the persistent disparity in tumor volume between EEA and TCA cohorts suggests that anatomy-driven selection has not yet been uniformly adopted. Finally, whether the pooled reporting of TSMs and PSMs as a single entity obscures clinically meaningful subtype differences remains unresolved [2,12].

The purpose of this systematic review is to compare contemporary outcomes of endoscopic endonasal and transcranial approaches for anterior midline skull base meningiomas in the nasoseptal flap era, with specific attention to (1) extent of resection, (2) postoperative visual and olfactory function, (3) cerebrospinal fluid leak and other perioperative complications, and (4) tumor recurrence—stratified, where data permit, by tumor subtype (olfactory groove vs. tuberculum sellae/planum sphenoidale meningiomas).

## 2. Materials and Methods

The research protocol for this systematic review was submitted to the International Prospective Registry of Systematic Reviews (PROSPERO) database and assigned the PROSPERO ID: CRD420251147118.

### 2.1. Study Identification

The PubMed, Scopus, and Embase databases were systematically searched in accordance with the Preferred Reporting Items for Systematic Reviews and Meta-Analyses (PRISMA) guidelines (Supplementary Data S1) [18]. Eligible studies were those presenting primary data and/or comparing outcomes between TCAs and EEAs for anterior cranial fossa meningiomas. The following Boolean search strategy was employed: (“Meningiomas” OR “Tuberculum Sellae Meningioma” OR “Olfactory Groove Meningioma” OR “Planum Sphenoidale Meningioma” OR “Anterior Skull Base Meningioma”) AND (“Endoscopy” OR “endoscopic” OR “endonasal” OR “endoscopic endonasal approach” OR “open transcranial” OR “craniotomy” OR “subfrontal approach” OR “pterional approach”) (Supplementary Data S2) [19]. The final database search was performed on 31 August 2025.

### 2.2. Study Selection Process

**Figure 1:**
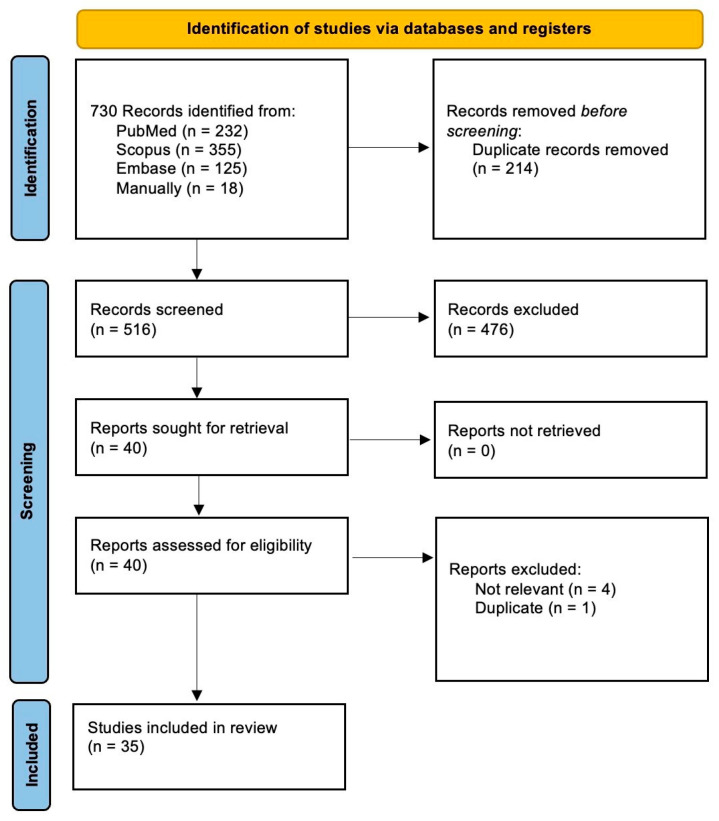
Flowchart showing an overview of the study selection process.

Following the removal of duplicate records using EndNote (version 20.2.1, Clarivate Analytics, Philadelphia, PA), two independent reviewers (U.S. and I.U.) screened the titles and abstracts for relevance. Full-text articles deemed potentially eligible were subsequently assessed by three independent reviewers (U.S., I.U., and Y.S.) to determine their suitability for inclusion in the final systematic review. Any disagreements were resolved through discussion and consensus among all reviewers. In addition, the reference lists of all included studies were examined to identify any further eligible publications. The screening and selection process is summarized in Figure 1.

### 2.3. Eligibility Criteria

Studies were eligible for inclusion if they reported on the surgical management of anterior midline skull base meningiomas—including olfactory groove, tuberculum sellae, and planum sphenoidale tumors—treated via either EEAs or TCAs, and met all of the following criteria: (1) publication between January 2010 and August 2025; (2) the EEA cohort utilized a vascularized nasoseptal flap for reconstruction; (3) full-text availability in English; (4) a total study population of at least 10 patients, with at least one treatment arm comprising 10 or more patients; and (5) reporting of at least one relevant clinical or surgical outcome. Studies were excluded if: (1) the transcranial cohort involved minimally-invasive keyhole approaches or combined endoscopic–microscopic techniques; (2) primary clinical data were not presented; or (3) the study focused exclusively on recurrent tumors.

### 2.4. Data Extraction

Data were independently extracted by two reviewers using a standardized protocol, with discrepancies resolved through discussion and consensus. Extracted variables included: study authors; year of publication; sample size; patient demographics (age and sex); presenting symptoms; tumor radiographic characteristics (volume, maximal diameter, optic and vascular involvement, and presence of peritumoral edema); extent of resection—categorized as gross total resection (GTR; Simpson grades I-III) or subtotal resection (STR; Simpson grades IV-V); postoperative visual and olfactory outcomes (new, improved, stable, or worsened); endocrine dysfunction; postoperative complications (CSF leak, meningitis, endocrine dysfunction, or mortality); and recurrence or progression rates. Data were extracted and pooled across studies using absolute event counts and corresponding denominators, with outcomes summarized as percentages. Continuous variables were recorded as means and standard deviations.

### 2.5. Data Analysis

Data analysis was conducted as part of a descriptive systematic review. Extracted data were pooled using absolute event counts and corresponding denominators and are presented as percentages within each surgical approach. Continuous variables, including tumor volume, maximal diameter, and patient age, were summarized as pooled means with standard deviations when available. When presented in alternative statistical formats, continuous variables were converted using the method described by Hozo et al. [20]. Given the heterogeneity across studies in design, patient selection, tumor characteristics, and outcome reporting, no formal meta-analysis or statistical comparisons were performed. Results were therefore synthesized narratively, with subgroup descriptions for OGMs, TSMs, and PSMs. Data aggregation and calculations were performed using R statistical software (version 4.3; R Foundation for Statistical Computing, Vienna, Austria).

### 2.6. Risk-of-Bias Assessment

Two authors (O.A., I.U.) assessed the risk of bias in the included studies using the National Institutes of Health (NIH) Quality Assessment Tool for Observational Cohort and Cross-sectional Studies [21]. The assessment involved rating studies on a scale of 0 for poor (0–4 out of 14 questions), i for fair (5–10 out of 14 questions), and ii for good (11–14 out of 14 questions). In cases where certain questions were not applicable (NA) or not reported (NR), these designations were used accordingly. For single-arm observational cohorts designated as case series in the original publications, the Joanna Briggs Institute (JBI) critical appraisal tool was utilized to evaluate the methodological quality of a study and determine the extent to which a study has addressed the possibility of bias in its design, conduct, and reporting methods [22]. When differences in assessment arose between the two authors, a third author was involved in mediating and resolving any disagreements, ensuring the integrity and consistency of the risk-of-bias evaluation process.

## 3. Results

### 3.1. Study Selection

Following full-text screening, we identified 35 articles that met our inclusion criteria and were included in our review. These articles included four prospective observational cohorts, 28 retrospective observational cohorts, and three case series [2,3,4,5,6,10,11,12,13,17,23,24,25,26,27,28,29,30,31,32,33,34,35,36,37,38,39,40,41,42,43,44,45,46,47]. Among these, five studies evaluated the EEA [11,12,17,23,24] and five studies compared the EEA and the TCA [5,6,13,25,26], while the remaining studies focused on TCA [2,3,4,10,27,28,29,30,31,32,33,34,35,36,37,38,39,40,41,42,43,44,45,46,47].

### 3.2. Endoscopic Endonasal Approach

A total of 10 studies, including 332 patients, were analyzed [5,6,11,12,13,17,23,24,25,26]. All included studies were observational cohort designs, both retrospective and prospective [23], and one was described as a case series [17]. The studies were conducted in the USA [5,6,12,17], China [24,26], Canada [11], Korea [13,25], and Vietnam [23] (Table 1). All studies focused on EEAs for anterior skull base meningiomas, including TSMs, PSMs, and OGMs, with one study specifically evaluating an expanded EEA technique [24]. Simpson grades were reported in only a few studies; therefore, the data could not be pooled based on that grading system. High rates of gross total resection (GTR) were consistently reported [5,6,12,23,24,26], and postoperative visual improvement was frequently observed [11,12,13,23,24,25,26]. Complication rates were generally low, although expanded EEA was associated with an increased risk of CSF leak and meningitis/infection [24].

#### 3.2.1. Demographics and Preoperative Characteristics of the Included Patients

The pooled analysis of 332 patients demonstrated a mean age of 54.6 years, with a strong female predominance of 76.9% (Table 2). Vision deterioration was the most common presenting symptom, reported in 282 of 316 patients, or 89.2%. Headache occurred in 39 of 197 patients (19.8%), while less common symptoms included seizure (7.7%), hyposmia/anosmia (20.5%), and pituitary dysfunction (11.5%). Incidental diagnosis was reported in 13 of 161 patients (8.1%).

Radiographically, the pooled mean tumor volume was 5.79 cm^3^, with studies reporting diameters only excluded from the estimate. Optic canal involvement was observed in 187 of 299 patients (62.5%), peritumoral edema in 18 of 109 (14.4%), and vascular involvement in 20 of 104 (19.2%). Henderson et al. [12] described five ACA and three internal carotid artery (ICA) encasements; however, the total number of affected patients could not be determined, so this study was excluded from the pooled analysis. Cortical cuff was reported only in Liu et al. [5], with five cases.

#### 3.2.2. Surgical Management and Postoperative Outcomes

**Olfactory Groove meningiomas**

Gross total resection (GTR) was achieved in 21 (91.3%) of 23 patients in the OGM group, with subtotal resection (STR) in two patients (8.7%). Preoperative and postoperative olfactory outcomes were assessed. Preoperatively, in the OGM group, 10 patients (43.5%) had normosmia, none had hyposmia, and 13 patients (56.5%) had anosmia. Postoperatively, anosmia was the most frequent olfactory dysfunction, observed in 19 of 21 patients (90.5%) in the OGM group.

Among the 23 patients with OGMs, postoperative complication data were available for 18 patients. The most common postoperative complication was meningitis or infection, occurring in three patients (16.7%), followed by CSF leakage in two patients (11.1%). No new postoperative endocrinopathies were reported. Mortality was observed in one patient (5.6%). Tumor recurrence occurred in two patients (8.7%) during a mean follow-up of 24.7 months (range 1–94 months).

**Tuberculum sellae/Planum sphenoidale meningiomas**

GTR was achieved in 281 of 309 patients (90.9%) in the TS/PS group, while STR was performed in 28 patients (9.1%). Olfactory function was assessed preoperatively in 311 patients, of whom 209 (67.2%) had documented normosmia. Postoperative olfactory status was reported for these same 209 patients with preoperative normosmia: normal olfaction was preserved in 190 (90.9%), while 17 (8.1%) deteriorated to hyposmia and two (1.0%) to anosmia. These figures therefore represent the proportion of patients with normal baseline olfaction in whom it was preserved after surgery, rather than any postoperative improvement in olfactory function. Visual outcomes were evaluated pre- and postoperatively. Preoperatively, 287 of 311 patients (92.3%) presented with impaired vision. Postoperatively, among patients with reported visual outcomes (*n* = 286), visual improvement was observed in 246 (86%), while 27 patients (9.4%) had stable impaired vision and 13 patients (4.5%) experienced worsened visual function. Among 311 patients, postoperative complications included CSF leakage in 31 patients (9.9%), meningitis or infection in 23 patients (7.4%), and endocrine dysfunction in eight patients (2.6%). Mortality occurred in one patient (0.3%). Tumor recurrence was reported in 12 patients (3.9%) across the included studies, with a mean follow-up of 29.1 months (range 0–131 months) (Table 3).

### 3.3. Transcranial Approaches

A total of 30 studies, encompassing 1534 patients [2,3,4,5,6,10,13,25,26,27,28,29,30,31,32,33,34,35,36,37,38,39,40,41,42,43,44,45,46,47], reporting on transcranial approaches for anterior skull base meningiomas were included in this section (Table 4). The studies, conducted between 2010 and 2025, originated from diverse regions, including Europe, Asia, and the Middle East. Most were retrospective cohort studies, with a few prospective and observational designs. Surgical approaches varied across studies and included anterior interhemispheric, bifrontal, frontal, interhemispheric, pterional, and subfrontal approaches, with multiple modified variants described in individual reports. The primary focus of these studies was on functional outcomes, particularly olfactory and visual preservation or recovery, as well as surgical safety and extent of resection.

#### 3.3.1. Demographics and Preoperative Characteristics of the Included Patients

The pooled analysis included 1534 patients who underwent a transcranial surgery for anterior skull base meningiomas. The mean patient age was 54.2 years, with a marked female predominance (73.1%). The most common presenting symptom was visual deterioration, affecting 67.1% of patients (1019/1519). Other frequent symptoms included headache 27.5% (330/1199), hyposmia/anosmia 22.3% (151/677), and altered mental status 27.2% (152/559). Seizure 11.2% (70/625), pituitary dysfunction 10.2% (21/205), and incidental discovery 9.1% (48/531) were less common.

Radiographic evaluation of included studies demonstrated a pooled mean tumor volume of 14.8 cm^3^. Optic canal involvement was observed in 46.3% of assessed cases (415/897), peritumoral edema in 33.1% (185/559), and vascular involvement was noted in 29.4% (119/405) (Table 5).

#### 3.3.2. Surgical Management and Postoperative Outcomes

**Olfactory Groove Meningiomas**

In the transcranial cohort of olfactory groove meningiomas, GTR was achieved in 329 of 358 patients (91.9%), whereas STR was performed in 29 patients (8.1%). Preoperative visual impairment was present in 59 patients (16.5%), and following surgery, visual function improved in 27 (7.5%), remained stable in 27 (7.5%), and worsened in 11 patients (3.1%).

Preoperative olfactory evaluation (*n* = 340) demonstrated normosmia in 181 patients (53.2%), hyposmia in 88 (25.9%), and anosmia in 71 (20.9%). Postoperatively, normosmia was preserved in 59 patients (17.4%), hyposmia was identified in 40 (11.8%), and anosmia occurred in 120 patients (35.3%).

Postoperative complications included cerebrospinal fluid (CSF) leakage in 38 of 358 patients (10.6%) and meningitis or infection in 12 patients (3.4%); no new endocrine dysfunction was reported. Mortality was reported in six of 358 patients (1.7%). Five were surgery-related—two within 30 days of surgery (Patel et al.) and three in the immediate postoperative period (pulmonary embolism, *n* = 1; cerebral edema, *n* = 2; Fountas et al.)—while one was non-surgical (post-discharge myocardial infarction; Zenga et al.). Tumor recurrence was documented in five patients (1.4%) across a mean follow-up period of 33.8 months (range 14.5–62 months).

**Tuberculum sellae/Planum sphenoidale meningiomas**

Among patients with tuberculum sellae or planum sphenoidale meningiomas, GTR was achieved in 1024 of 1176 cases (87.1%), whereas 150 patients (12.8%) underwent STR. Preoperatively, visual impairment was present in 940 of 1174 patients (80.0%). Among patients with reported postoperative visual outcomes (*n* = 966), improvement occurred in 589 patients (61%), visual status remained stable in 278 (28.8%), and it worsened in 99 patients (10.2%).

Olfactory assessment (*n* = 1016) showed preoperative normosmia in 465 patients (45.8%), hyposmia in five (0.5%), and anosmia in 18 (1.8%). Postoperatively, normosmia was preserved or restored in 694 patients (68.3%), while hyposmia and anosmia occurred in 67 (6.6%) and 36 (3.5%), respectively.

Postoperative complications included CSF leakage in 13 of 1178 patients (1.1%), meningitis or infection in 33 (2.8%), and new-onset endocrine dysfunction in 71 patients (6.0%). Mortality was reported in nine of 1178 patients (0.8%). Only one death was directly surgery-related (intraoperative internal carotid artery rupture; Song et al.); the remaining eight were non-surgical, comprising one death of unspecified cause (Kong et al.) and seven deaths from comorbidities during follow-up, for which the authors explicitly reported no surgical mortality (Dokponou et al.). Recurrence was reported in 53 of 1029 patients (5.2%) over a mean follow-up of 40.6 months (range 12–62 months) (Table 6).

### 3.4. Quality Assessment of Included Studies

We utilized JBI and NIH tools to assess the quality of the 35 studies included in this analysis (three case series with JBI [17,44,46]; remaining 32 cohort studies with NIH). All three case series were rated as good quality. Among the observational studies, one study [26] was rated as fair quality, and the rest were rated as high quality [2,3,4,5,6,10,11,12,13,17,23,24,25,27,28,29,30,31,32,33,34,35,36,37,38,39,40,41,42,43,44,45,46,47]. Further details regarding the study quality analysis are displayed in Supplementary Tables S1 and S2.

## 4. Discussion

The optimal approach to anterior midline meningiomas remains a subject of active clinical and academic debate within modern skull base surgery, with no well-established guidelines to direct approach selection. OGMs, PSMs, and TSMs pose distinct surgical challenges due to their proximity to the optic apparatus, the ACA complex, and the olfactory pathways, as well as the frequent presence of hyperostosis and variable lateral extension. The evolution of the EEA, particularly following widespread adoption of NSF reconstruction, necessitates a reappraisal of its role relative to established TCA corridors, not only in terms of the extent of resection, but also with respect to functional outcomes and complication profiles. This review reframes surgical decision-making by demonstrating that, in the modern NSF era, approach selection should be driven less by historical concerns over CSF leak and more by tumor subtype-specific anatomical and functional considerations.

Prior to widespread NSF adoption, CSF leak rates following EEAs for anterior skull base tumors frequently exceeded 20–30% [7,8]. The introduction of the NSF reduced this complication substantially and established a reliable reconstructive strategy for extended endoscopic endonasal approaches [9]. Several contemporary series have demonstrated leak rates below 10% with multilayer NSF-based closure [13,17,24]. However, although several comparative syntheses exist [1,48,49], none has specifically isolated the contemporary nasoseptal flap era as the defining inclusion window. This systematic review addresses that gap by analyzing 35 studies comprising 1866 patients from 2010 to 2025.

**Figure 2:**
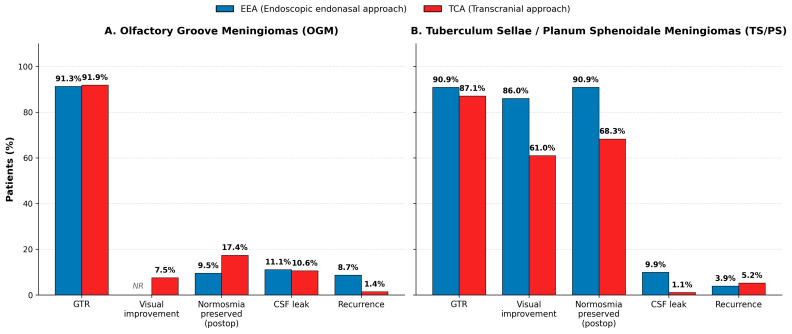
Comparative pooled outcomes of EEAs and TCAs for anterior midline skull base meningiomas, stratified by tumor subtype. (**A**) Olfactory groove meningiomas; (**B**) tuberculum sellae and planum sphenoidale meningiomas. NR, not reported. EEA, endoscopic endonasal approach; TCA, transcranial approach; GTR, gross total resection; CSF, cerebrospinal fluid.

Across included studies, both EEAs and TCAs achieved high gross total resection (GTR) rates. For olfactory groove meningiomas (OGMs), GTR was comparable between EEAs (91.3%) and TCAs (91.9%), and similarly high for TS/PS meningiomas (EEA 90.9%, TCA 87.1%). These comparable results support the principle that, in carefully selected cases, either approach can yield effective tumor control. Despite this oncologic equivalence, functional outcomes differed significantly (Figure 2). Among patients with reported postoperative visual outcomes, EEAs provided markedly superior visual improvement for TS/PS meningiomas (86%) compared with TCAs (61%). This advantage is anatomically grounded: the direct inferomedial corridor permits early optic canal decompression with minimal direct manipulation of the optic nerves and chiasm and preserves the delicate inferomedial vascular supply to the optic apparatus—small perforators that are particularly vulnerable during transcranial dissection along the superior and lateral aspects of the optic nerve [6,25,50]. A particularly relevant anatomical advantage of EEAs is their capacity for bilateral optic canal decompression through a single midline corridor. In contrast, standard unilateral pterional or subfrontal transcranial approaches provide direct access to the ipsilateral optic canal, with contralateral decompression requiring additional dissection or approach modification. Given that bilateral optic canal invasion is present in a substantial proportion of TSMs—with optic canal involvement reported in 62.5% of EEA patients in our series—this bilateral decompression capability may confer meaningful advantages in patients with symmetric or bilateral visual compromise. The endonasal trajectory also enables immediate exposure of the tuberculum and planum and avoids manipulation of the anterior cerebral vasculature until after decompression has been achieved [6,12]. This anatomical rationale is consistent with foundational endoscopic endonasal skull base series [50]. However, the visual advantage of EEAs over TCAs for TSMs may be partially attributable to inconsistent application of optic canal decompression techniques in transcranial series. Early optic canal unroofing, whether performed intradurally or extradurally, has been shown to significantly improve visual outcomes following transcranial resection of TSMs. Nozaki et al. demonstrated that early unroofing of the optic canal during transcranial surgery was associated with superior visual recovery, suggesting that the timing and completeness of canal decompression—rather than the surgical corridor alone—is a primary determinant of visual outcome [14]. Otani et al. further described the selective extradural anterior clinoidectomy (SEAC) technique as an effective adjunct during pterional approaches for TSMs, particularly in cases with tumor extension into the optic canal, sella, or hypothalamus, reporting visual improvement in 78.1% of patients with complete tumor removal in 87.5% [15]. More recently, Sadigh et al. reported that routine extradural optic canal decompression performed by skull base-trained surgeons was associated with improved visual outcomes across both TSMs and anterior clinoid process meningiomas [16]. These findings suggest that when transcranial approaches are combined with systematic optic canal unroofing—whether via extradural clinoidectomy or intradural decompression—the visual outcome gap between TCAs and EEAs may narrow considerably. The superiority of EEAs in our pooled analysis may therefore partly reflect differences in the routine adoption of canal decompression across included TCA series rather than an inherent limitation of the transcranial corridor itself. In contrast, postoperative olfactory outcomes strongly favored transcranial techniques. In the OGM group, postoperative anosmia occurred in 90.5% of EEA cases versus 35.3% of TCA cases. This difference reflects the fundamental anatomical constraints of EEAs: removal of the cribriform plate disrupts olfactory filaments, making anosmia nearly inevitable except in patients who already present with complete loss of smell. Transcranial approaches—particularly unilateral subfrontal, pterional, and orbitofrontal variants—allow preservation of the cribriform plate and minimize traction on the olfactory tracts, thereby supporting better olfactory preservation. The technical nuances of these approaches, such as limited frontal lobe elevation and tailored dural opening, also contribute to these superior olfactory outcomes [3,4,10,38,44,45].

Tumor size significantly influenced outcomes and approach selection across the included studies. EEA cohorts predominantly consisted of smaller tumors, whereas TCA series more frequently included large or giant lesions, particularly among OGMs. Large tumors often require extensive drilling of hyperostotic bone, management of lateral extension, and dissection around vascular encasement: factors that are traditionally considered more amenable to correction via a transcranial route. Consistent with this selection pattern, radiographic markers of tumor complexity, particularly vascular involvement (29.4% in TCA vs. 19.2% in EEA) and peritumoral edema (33.1% in TCA vs. 16.5% in EEA), were more frequently observed in TCA cohorts. These features may have contributed to baseline differences between groups, potentially influencing the observed differences in functional outcomes and complication profiles between approaches. This substantial difference in tumor volume represents the dominant confounder in any cross-approach comparison and may, in large part, account for the stronger visual outcomes reported for EEAs: the EEA cohorts comprised smaller, more midline tumors that are inherently more amenable to favorable visual outcomes regardless of the surgical corridor. These pooled differences should therefore be interpreted as reflecting case selection rather than the intrinsic superiority of either approach. Heterogeneous reporting of tumor characteristics such as degree of hyperostosis, peritumoral edema, and optic canal invasion further complicates direct cross-study comparisons.

Postoperative CSF leak remains a defining complication of the endonasal approach. In our analysis, the leak rate among OGMs was 11.1% and 9.9% for TS/PS meningiomas. Expanded EEA techniques, as reported by Yu et al., were associated with higher rates of meningitis, mirroring CSF leak trends [24]. Meanwhile, CSF leak rates after TCA were substantially lower for TS/PS meningiomas (1.1%), reflecting the advantage of traditional dural closure techniques and the absence of high-flow nasal cavity communication, although these rates were comparable to EEAs for OGMs (10.6%), aligning with prior reports [1]. The elevated leak rate in OGMs treated transcranially is likely related to the extensive drilling required to remove hyperostotic anterior skull base bone and the large dural defects created during reconstruction. These anatomical and technical variables underscore that CSF leak risk depends more on the nature of the tumor and the defect than on the approach itself [33].

Recurrence rates were low and similar across techniques. In EEA cohorts, recurrence occurred in 8.7% of OGMs and 3.9% of TS/PS meningiomas; in TCA cohorts, 1.4% and 5.2%, respectively. The slightly higher recurrence in TCA TS/PS meningioma cases may reflect a higher proportion of large tumors or those with vascular encasement, although reporting inconsistency limits definitive interpretation.

Taken together, these findings support a nuanced, anatomy-driven framework for operative decision-making. Tumors treated with EEAs were typically smaller, more midline in location, and more frequently demonstrated optic canal involvement, a constellation of features for which an EEA is generally considered most advantageous, particularly in TS/PS meningiomas presenting with predominant visual symptoms. Patients with severe preoperative visual impairment may particularly benefit from the early decompression afforded by the endonasal route. Conversely, tumors managed via a TCA tended to be larger and more complex, a profile more commonly encountered in olfactory groove meningiomas, for which transcranial surgery is often preferred when preservation of olfaction represents an important patient priority. They also provide more versatile access for large or giant tumors, significant lateral extension, extensive hyperostosis, or vascular involvement. Importantly, preoperative olfactory status influences the aggressiveness of resection in both approaches: patients with complete anosmia may tolerate more extensive drilling or resection of the cribriform region.

## 5. Limitations

Limitations of our analysis include those inherent to systematic reviews in general; the quality is dependent upon the quality of evidence of the studies presented, the majority of which consisted of retrospective cohort studies. These are prone to multiple different types of bias, particularly selection and reporting bias. Furthermore, key tumor characteristics—such as volume, hyperostosis, lateral extension, and optic canal or vascular involvement—were inconsistently reported, limiting direct comparison between approaches. In particular, the aggregate-level outcome reporting of most included studies precluded a formal volume-matched comparison between approaches; the substantial difference in mean tumor volume between cohorts (TCA 14.8 vs. EEA 5.79 cm^3^) therefore remains an important confounder that pooled descriptive data cannot fully resolve. Olfactory outcomes were assessed variably, ranging from subjective descriptions to formal testing. This significant heterogeneity in outcome measures and patient population precluded any formal meta-analysis of studies with regard to these outcomes. Reconstruction techniques and surgeon experience differed widely across centers, influencing complication rates, particularly CSF leakage. Finally, heterogeneity in follow-up duration reduced the ability to reliably assess long-term recurrence. Additionally, tuberculum sellae and planum sphenoidale meningiomas were analyzed as a combined subgroup (TS/PS) throughout this review, consistent with the reporting conventions of the majority of included studies. While these two entities differ in their anatomical origin and clinical presentation—TSMs being more commonly associated with optic canal involvement and early visual dysfunction, and PSMs more often presenting with frontal lobe symptoms—the insufficient number of studies reporting outcomes exclusively for PSMs precluded independent subgroup analysis. Future studies with dedicated PSM cohorts would enable more granular subgroup comparisons.

This review was deliberately structured as a two-arm comparison of EEAs (with nasoseptal flap reconstruction) and conventional open transcranial approaches; the supraorbital keyhole corridor was therefore not included. The keyhole approach represents a distinct minimally invasive paradigm with its own selection criteria and outcome profile, and pooling it with conventional craniotomy data would have confounded the intended comparison. A dedicated three-arm comparison (EEA vs. keyhole vs. conventional transcranial approach) represents an important direction for future systematic reviews.

Our analysis also has several strengths. Because we were able to pool data from over 1800 patients across 35 studies, we were able to characterize and report rare complications and study tumors of varying complexity. For example, the ability to classify tumors by markers of operative and anatomic complexity, such as tumor size, presence of peritumoral edema, and vascular involvement, provides important context when interpreting results related to functional outcome. Additionally, our inclusion of the literature beyond 2010 provides a contemporary understanding of functional outcomes in the setting of advanced NSF techniques. As noted above, heterogeneity across studies—including variability in imaging assessment, surgical technique, reconstruction methods, and surgeon experience—must temper interpretation. Nonetheless, the consistent patterns observed across nearly two thousand patients underscore that functional outcomes diverge not because one approach is universally superior, but because each aligns differently with specific anatomical configurations.

In summary, both EEAs and TCAs achieve excellent tumor control for anterior midline skull base meningiomas but differ in their functional strengths. Understanding these distinctions enables personalized, anatomy-informed surgical planning that optimizes visual and olfactory outcomes while minimizing complications.

## 6. Conclusions

This systematic review supports three key conclusions: (1) In the nasoseptal flap era, EEAs and TCAs achieve comparable gross total resection rates (≈90%) for anterior midline skull base meningiomas; oncologic efficacy is therefore no longer a discriminating factor between the two corridors. (2) The EEA is associated with superior postoperative visual improvement in TS/PS meningiomas (86% vs. 61%), attributable to early optic canal decompression and minimal manipulation of the optic apparatus; however, this advantage is confounded by selection bias, as EEA cohorts comprised smaller, more midline tumors, and may narrow when transcranial approaches systematically incorporate early optic canal unroofing. (3) The TCA remains the preferred corridor for olfactory preservation in olfactory groove meningiomas (postoperative anosmia 35.3% vs. 90.5% with EEAs) and for large, laterally extensive, or vascularly involved tumors that exceed the safe operative window of the endonasal route. Approach selection should therefore be individualized according to tumor subtype, size, lateral extension, optic canal involvement, and the patient’s functional priorities, rather than applied uniformly. Prospective, volume-matched comparative studies are needed to refine evidence-based approach selection and to disentangle the effect of surgical corridor from that of case selection.

## Figures and Tables

**Table 1:** Characteristics of studies reporting endoscopic endonasal approach outcomes (comparative studies were included if EEA-specific outcomes were extractable).

Authors	Year	Country	Study Design	Approach	Aim	Conclusions
Bander et al. [6]	2017	USA	Retrospective cohort Sample size (EEA/TCA): 17/15 Mean age (EEA/TCA): 54.3 ± 14.3/55.7 ± 12.9 years Mean follow-up duration (EEA/TCA): 25.1/37.0 months	EEA vs. TCA	To compare TCA and EEA outcomes in patients with TS/PS meningiomas suitable for either approach.	EEA achieved similar resection with better visual and brain outcomes, supporting its use in select TS/PS meningiomas.
Henderson et al. [12]	2023	USA	Retrospective cohort Sample size: 47 Mean age: 55 ± 16 years Median follow-up duration (range): 28.5 (0.1–131) months	EEA	To assess whether distinguishing PSMs from TSMs impacts EEA outcomes.	Outcomes were similar for both, slightly favoring TSMs. Lateral tumor extension, rather than origin, was a stronger predictor of outcome.
Kong et al. [25]	2019	Korea	Retrospective cohort Sample size (EEA/TCA): 84/194 Mean age (EEA/TCA): 54.2 ± 13.6/53.7 ± 11.0 years Mean follow-up duration (range): 28.0 (3.1–70.6) months	EEA vs. TCA	To analyze the key anatomical features of TSM and compare the endoscopic endonasal and transcranial surgical approaches.	The study favors EEA for better visual outcomes with acceptable complications, though TCA remains effective for TSM.
Liu et al. [5]	2018	USA	Retrospective cohort Sample size (EEA/TCA/Combined): 5/15/8 Mean age (EEA/TCA/Combined): 51.1 (28–58) / 52.1 (34–66) / 52.9 (15–83) years Mean follow-up duration (range): 14.5 (1–76) months	EEA vs. TCA vs. Combined	To analyze factors influencing approach selection and surgical outcomes in OGMs.	EEA is best suited for smaller OGMs with preexisting olfactory loss and serves as an adjunct to transbasal approaches in recurrent cases.
Qian et al. [26]	2022	China	Retrospective cohort Sample size (EEA/TCA): 34/78 Mean age (EEA/TCA): 52.2 (10.1)/ 50.5 (11.7) years Mean follow-up duration (range): 20.5 (3–36) months	EEA vs. TCA	To compare surgical outcomes and complications of TCAs and EEAs for TSMs in order to define optimal surgical management principles.	Both TCAs and EEAs effectively remove TSMs, but EEAs offer superior visual improvement or stabilization compared to TCAs.
Schroeder et al. [11]	2025	Canada	Retrospective cohort Sample size: 24 Mean age: 51.0 years Mean follow-up duration (days): 1906.1 days	EEA	To evaluate clinical outcomes and volumetric changes after EEA for TSMs and PSMs.	EEAs achieved favorable clinical and radiographic outcomes, with high GTR rates and minimal morbidity.
Song et al. [13]	2018	Korea	Retrospective cohort Sample size (EEA/TCA): 44/40 Mean age (EEA/TCA): 52.7 (26–76)/54.4 (24–74) years Median follow-up duration (EEA/TCA): 27/43.5 months	EEA vs. TCA	To compare resection extent, recurrence, and visual outcomes between EEAs and TCAs for TSMs.	EEA provided better visual outcomes and is increasingly favored for pure TSMs; GTR rates were similar, but recurrence patterns differed.
Truong et al. [23]	2022	Vietnam	Prospective cohort Sample size: 26 Mean age (range): 56 (30–72) years Mean follow-up duration (range): 28 (3–60) months	EEA	To evaluate the effectiveness of EEAs for ASB meningiomas and determine factors affecting surgical outcomes.	EEA is a safe and effective alternative to open craniotomy for ASB meningiomas in selected patients, though postoperative CSF leaks and meningitis remain potential drawbacks.
Wang et al. [17]	2024	USA	Case series Sample size: 13 Mean age (range): 60.4 (36.1–82.6) years Mean follow-up duration (range): 31.6 (1–94) months	EEA	To review OGM resections by a single surgeon, focusing on surgical nuances, patient selection, and outcomes.	EEA is an effective and safe method for OGM resection, with outcomes and complication rates comparable to transcranial approaches.
Yu et al. [24]	2021	China	Retrospective cohort Sample size: 40 Mean age (range): 58.3 (42–73) years Median follow-up duration (range): 15 (5–57) months	Expanded EEA	To evaluate outcomes of expanded EEAs in TSM treatment.	Expanded EEAs may provide superior visual outcomes and higher GTR rates compared to craniotomy, though with increased CSF leak risk.

ASB, Anterior Skull Base; CSF, Cerebrospinal Fluid; EEA, Endoscopic Endonasal Approach; Expanded EEA, Expanded Endoscopic Endonasal Approach; GTR, Gross Total Resection; OGM, Olfactory Groove Meningioma; PSM, Planum Sphenoidale Meningioma; TCA, Transcranial Approach; TSM, Tuberculum Sellae Meningioma; TS/PS, Tuberculum Sellae/Planum Sphenoidale.

**Table 2:** Summary of preoperative symptoms pooled data from the included endoscopic endonasal approach studies.

Category	Event (*n*)	Total (*N*)	(%)
**Demographics (*n* = 332)**			
Mean age (weighted) (Years)	54.6	332	–
Male	67	290	23.1%
Female	223	290	76.9%
**Presenting Symptoms**			
Vision deterioration	282	316	89.2%
Headache	39	197	19.8%
Seizure	3	39	7.7%
Pituitary dysfunction	3	26	11.5%
Hyposmia/anosmia	8	39	20.5%
Incidental diagnosis	13	161	8.1%
**Tumor Radiographic Characteristics**			
Mean tumor volume (cm^3^)	5.79	114	–
Mean tumor diameter (mm)	23.8	113	–
Optic canal involvement	48	107	62.5%
Vascular involvement	20	104	19.2%
Peritumoral edema	18	109	16.5%

**Table 3:** Outcomes of endoscopic endonasal surgery for anterior midline skull base meningiomas by tumor location.

Variable	OG			TS + PS		
	Event (*n*)	Total (*N*)	(%)	Event (*n*)	Total (*N*)	(%)
**Extent of Resection**						
GTR	21	23	91.3%	281	309	90.9%
STR	2	23	8.7%	28	309	9.1%
**Visual Outcomes**						
Preoperative						
Impaired	NR	NR	-	287	311	92.3%
Postoperative						
Improved	NR	NR	-	246	286	86%
Stable (impaired)	NR	NR	-	27	286	9.4%
Worsened	NR	NR	-	13	286	4.5%
**Olfactory Outcomes**						
Preoperative						
Normosmia	10	23	43.5%	209	311	67.2%
Hyposmia	0	23	0%	0	209	0%
Anosmia	13	23	56.5%	0	209	0%
Postoperative						
Normosmia	2	21	9.5%	190	209	90.9%
Hyposmia	0	21	0%	17	209	8.1%
Anosmia	19	21	90.5%	2	209	1%
**Postoperative Complications**						
CSF leakage	2	18	11.1%	31	311	9.9%
Meningitis/infection	3	18	16.7%	23	311	7.4%
Endocrine dysfunction	0	18	0%	8	311	2.6%
Mortality						
Surgical (perioperative)	1	18	5.6%	1	311	0.3%
Non-surgical	0	18	0%	0	311	0%
Recurrence	2	23	8.7%	12	311	3.9%
Follow-up duration (mean (range))	24.7 (1–94)	23	-	29.1 (1–131)	311	-

CSF, cerebrospinal fluid; GTR, gross total resection; NR, not reported; OG, olfactory groove; PS, planum sphenoidale; STR, subtotal resection; TS, tuberculum sellae; TS + PS, tuberculum sellae/planum sphenoidale.

**Table 4:** Characteristics of studies reporting transcranial approach outcomes (comparative studies were included if TCA-specific outcomes were extractable).

Authors	Year	Country	Study Design	Approach	Aim	Conclusions
Aftahy et al. [2]	2020	Germany	Retrospective cohort Sample size: 88 Mean age (range): 60 (50–73) years Median follow-up duration (range): 15.5 (0–112) months	Frontal interhemispheric; unilateral subfrontal, bifrontal, pterional, frontolateral, supraorbital	To propose a decision-making algorithm for selecting surgical approaches.	Median approaches suited OGMs; lateral approaches favored TS/PS meningiomas with good visual outcomes.
Bander et al. [6]	2017	USA	Retrospective cohort Sample size (EEA/TCA): 17/15 Mean age (EEA/TCA): 54.3 ± 14.3/55.7 ± 12.9 years Mean follow-up duration (EEA/TCA): 25.1/37.0 months	EEA vs. TCA	To compare TCA and EEA outcomes in patients with TS/PS meningiomas suitable for either approach.	EEA achieved similar resection with better visual and brain outcomes, supporting its use in select TS/PS meningiomas.
Costanzo et al. [30]	2023	Italy	Retrospective cohort Sample size: 22 Mean age: 59.5 ± 12.6 years Follow-up duration: 6 months	Pterional and anterior interhemispheric approach	To compare surgical outcomes of two approaches.	Both were effective; approach choice should be based on surgeon expertise.
Curey et al. [31]	2012	France	Retrospective cohort Sample size: 20 Mean age: 59.1 ± 11.1 years Mean follow-up duration: 56.3 ± 34 months	Superior interhemispheric approach	This study aimed to assess visual outcomes and surgical risks following TSm removal via the superior interhemispheric approach.	The superior interhemispheric approach improves TSm-related vision loss but often causes olfactory decline.
Dedeciusova et al. [10]	2020	Czech Republic	Prospective Cohort Sample size: 13 Mean age (range): 59 (40–80) years Mean follow-up duration (range): 26 (13–54) months	Unilateral approach	To assess olfactory function after anterior skull base resections.	Olfactory assessment is essential; outcomes depended on preoperative factors and tumor extension.
Dokponou et al. [43]	2024	Morocco	Retrospective cohort Sample size: 91 Mean age: 51 ± 13.6 years Median follow-up duration (range): 12 (10–63) months	Frontolateral and pterional approach	This study sought to detail the surgical treatment and long-term outcomes of PSMs.	The pterional approach enables gross-total resection of jugum sphenoidale meningiomas and reduces the likelihood of recurrence.
Duan et al. [33]	2024	China	Retrospective cohort Sample size: 208 Mean age (range): 50.5 (19–78) years Median follow-up duration (range): 63 (12–152) months	Pterional, subfrontal, lateral supraorbital	To analyze prognostic factors for visual recovery after TSM resection.	Favorable outcomes linked to age, tumor size, GTR, and dimensional ratio.
Duangprasert et al. [32]	2025	Thailand and Japan	Retrospective cohort Sample size: 24 Mean age (range): 51.5 (36–78) years Mean follow-up duration (range): 27.3 (4–73) months	Bifrontal and bifrontal transbasal approach	To evaluate outcomes of tumors with optic canal invasion.	Optic canal exploration improved resection, vision, and reduced recurrence risk.
Emmez et al. [44]	2021	Turkiye	Case series Sample size: 18 Mean age (range): 52 (36–75) years Mean follow-up duration (range): 36 (2–60) months	Unilateral frontotemporal	To present rationale and outcomes of unilateral frontotemporal approach for OGM.	Achieved high resection rates with low complications.
Engelhardt et al. [29]	2018	France	Retrospective cohort Sample size: 20 Mean age (range): 50.5 (43.5–59.6) years Median follow-up duration (range): 5.6 (1.45–7.3) years	Controlateral approach	To present a TSM surgical approach targeting inferomedial optic nerve involvement and assess outcomes and complications.	This approach enabled safer optic nerve mobilization, preserved vascular supply, and provided direct access to its inferomedial side.
Fountas et al. [3]	2018	Greece, Germany, Romania	Retrospective cohort Sample size: 78 Mean age (range): 63.3 (20–78) years Mean follow-up duration (years)(range): 5.6 (2–15)	Bilateral subfrontal, unilateral frontal, unilateral pterional approach	To evaluate the surgical and neuropsychological outcomes of OGMs to guide the development of effective surgical strategies.	Tailored surgical strategies are needed to reduce complications and recurrence in OGMs, while the role of less invasive endoscopic approaches remains unclear.
Grutza et al. [41]	2024	Germany	Retrospective cohort Sample size: 71 Mean age: 56.9 ± 14.3 years Mean follow-up duration: 32.9 ± 33.7 months	Pterional approach	To evaluate the outcomes of the pterional approach for TSMs, focusing on resection rates, neurological and visual outcomes, and surgical complications.	TSMs can be effectively resected via the pterional approach with low complication risk and good visual outcomes, though larger studies are needed to confirm these results.
Hannequin et al. [38]	2015	France	Prospective longitudinal study Sample size: 10 Mean age: 47.5 ± 9.3 years	Superior interhemispheric approach	To evaluate postoperative olfactory outcomes following the IH approach for anterior skull base meningiomas.	Olfactory function was mostly preserved; decline was related to tumor size and invasiveness.
Karsy et al. [28]	2017	India	Retrospective cohort Sample size: 49 Mean age: 53.2 ± 14.0 years Mean follow-up duration: 42.3 ± 45.4 months	Frontotemporal; bifrontal	To evaluate the outcomes of transcranial resection for tuberculum sellae meningiomas and assess its suitability compared to less invasive approaches.	The frontotemporal approach for TSMs achieves GTR, manageable complications, and good visual outcomes, making it a favorable option.
Kim et al. [36]	2022	Korea	Retrospective cohort Sample size: 74 Mean age: 54.4 years Median follow-up duration (range): 63 (2–185) months	Contralateral subfrontal approach	To report long-term outcomes and experience with the contralateral subfrontal approach for TSMs.	The contralateral subfrontal approach allows safe TSM removal and visual improvement with minimal complications.
Kong et al. [25]	2019	Korea	Retrospective cohort Sample size (EEA/TCA): 84/194 Mean age (EEA/TCA): 54.2 ± 13.6/53.7 ± 11.0 years Mean follow-up duration (range): 28.0 (3.1–70.6) months	EEA vs. TCA	To analyze the key anatomical features of TSM and compare the endoscopic endonasal and transcranial surgical approaches.	The study favors EEA for better visual outcomes with acceptable complications, though TCA remains effective for TSM.
Leclerc et al. [40]	2022	France	Retrospective cohort Sample size: 50 Mean age (range): 54.3 (25–78) years Mean follow-up duration: 32.4 ± 20.2 months	Unilateral subfrontal, interhemispheric	To identify predictors of visual improvement after TSM surgery.	Early surgery improved chances of visual restoration.
Li-Hua et al. [37]	2011	China	Retrospective cohort Sample size: 67 Mean age (range): 48.7 (28–76) years Mean follow-up duration (range): 29.3 (6–48.5) months	Frontolateral approach, fronto-orbital approach	To assess visual outcomes in TSM patients undergoing microsurgical resection via frontolateral or fronto-orbital approaches with optic canal unroofing.	The frontolateral approach allows safe, total TSM resection with minimal morbidity and improved vision, aided by optic nerve decompression and canal unroofing.
Liu et al. [5]	2018	USA	Retrospective cohort Sample size (EEA/TCA/Combined): 5/15/8 Mean age (EEA/TCA/Combined): 51.07 (28–58)/52.13 (34–66)/52.88 (15–83) years Mean follow-up duration (range): 14.5 (1–76) months	EEA vs. TCA vs. Combined	To analyze factors influencing approach selection and surgical outcomes in OGMs.	EEA is best suited for smaller OGMs with preexisting olfactory loss and serves as an adjunct to transbasal approaches in recurrent cases.
Patel et al. [4]	2019	UK	Retrospective cohort Sample size: 50 Mean age (range): 62.06 (23–84) years Mean follow-up duration (range): 59.25 (17–157) months	Bifrontal transbasal with removal of supraorbital bar, bifrontal transbasal with modified supraorbital osteotomy, bifrontal interhemispheric, frontal craniotomy	To evaluate how particular imaging characteristics influence outcomes after transcranial resection of large or endoscopically challenging OGMs, providing a benchmark for comparing endoscopic results.	Transcranial resection yields good results even for large or complex OGMs, and features that limit endoscopic surgery do not adversely impact outcomes with TCA.
Puppa et al. [39]	2015	Italy	Retrospective cohort Sample size: 43 Mean age (range): 63 (38–81) years Mean follow-up duration (range): 41(3–77) months	TCA	To determine the safety and effectiveness of open transcranial resection for midline anterior cranial base meningiomas < 35 mm.	Surgery was safe and effective, particularly in younger patients and those without preoperative visual loss.
Qian et al. [26]	2022	China	Retrospective cohort Sample size (EEA/TCA): 34/78 Mean age (EEA/TCA): 52.2 (10.1)/50.5 (11.7) years Mean follow-up duration (range): 20.5 (3–36) months	EEA vs. TCA	To compare surgical outcomes and complications of TCA and EEA for TSM in order to define optimal surgical management principles.	Both TCA and EEA effectively remove TSMs, but EEA offers superior visual improvement or stabilization compared to TCA.
Song et al. [13]	2018	Korea	Retrospective cohort Sample size (EEA/TCA): 44/40 Mean age (EEA/TCA): 52.7 (26–76)/ 54.4 (24–74) years Median follow-up duration (EEA/TCA): 27/43.5 months	EEA vs. TCA	To compare resection extent, recurrence, and visual outcomes between EEAs and TCAs for TSMs.	EEA provided better visual outcomes and is increasingly favored for pure TSMs; GTR rates were similar, but recurrence patterns differed.
Troude et al. [34]	2021	France	Retrospective cohort Sample size: 94 Mean age (range): 55 (21–86) years Mean follow-up duration (range): 59 (2–210) months	Ipsilateral and contralateral approach	To compare long-term visual and olfactory outcomes and recurrence rates in TSM patients operated via ipsilateral versus contralateral approaches.	Contralateral resection of TSMs offers improved visual results and tumor control, but with a higher risk of olfactory dysfunction.
Wilk et al. [47]	2016	Poland	Retrospective cohort Sample size: 18 Mean age (range): 50.5 (30–73) years Mean follow-up duration: 20.55 months	Unilateral subfrontal approach	To evaluate visual and endocrinological outcomes after TSM surgery.	Subfrontal resection improved vision while preserving pituitary function.
Xiao et al. [46]	2021	China	Case series Sample size: 10 Mean age: 54 ± 3.7 years Follow-up duration: 6 months	Unilateral subfrontal approach	To examine the surgical technique, outcomes, and complications of the unilateral subfrontal approach for giant tuberculum sellae meningiomas, supported by a literature review.	Unilateral forehead-bottom craniotomy is effective for giant TSMs, with EC glue and fascia flap preventing frontal sinus complications, and optic canal unroofing significantly improving vision.
Xu et al. [42]	2019	China	Prospective observational study Sample size: 54 Mean age (range): 53.2 (34–73) years Mean follow-up duration (range): 39.5 (16–64) months	Extended bifrontal approach	To describe a small extended bifrontal approach and its outcomes in MASB meningiomas.	Provided good exposure and safe resection with minimal morbidity.
Yılmaz et al. [35]	2025	Turkiye	Retrospective cohort Sample size: 16 Mean age: 62.1 years Follow-up duration (range): 2 (1 month–3) years	Unilateral extended pterional approach	To assess outcomes of extended pterional craniotomy for large OGMs.	Safe and effective for giant OGMs with minimal complications.
Zenga et al. [45]	2020	Italy	Retrospective cohort Sample size: 50 Mean age: 61.3 years Mean follow-up duration (range): 62 (4–103) months	Trans-frontal sinus approach	To demonstrate a surgical approach that combines the advantages of both endoscopic and transcranial techniques.	Although no consensus exists on the optimal OGM surgery, this study suggests the trans-frontal sinus approach as a viable alternative, combining benefits of endoscopic and traditional transcranial methods.
Zhou et al. [27]	2015	China	Retrospective cohort Sample size: 56 Mean age (range): 42.5 (21–69) years Mean follow-up duration: 27.5 ± 7.2 months	Unilateral subfrontal, lateral frontobasal, frontotemporal	To assess visual outcomes after microsurgical resection of TSMs.	Visual improvement occurred in most patients; morbidity was low with no mortality.

EEA, Endoscopic Endonasal Approach; GTR, Gross Total Resection; IH, Interhemispheric; MASB, Midline Anterior Skull Base; OGM, Olfactory Groove Meningioma; PSM, Planum Sphenoidale Meningioma; PSMs, Planum Sphenoidale Meningiomas; TCA, Transcranial Approach; TSM, Tuberculum Sellae Meningioma; TS/PS, Tuberculum Sellae/Planum Sphenoidale.

**Table 5:** Summary of preoperative symptoms pooled data from the included transcranial approach studies.

Category	Event	Total (*n*)	(%)
**Demographics (*n* = 1534)**			
Mean age	54.2	1534	-
Male	403	1497	26.9%
Female	1094	1497	73.1%
**Presenting Symptoms**			
Vision deterioration	1019	1519	67.1%
Headache	330	1199	27.5%
Hyposmia/anosmia	151	677	22.3%
Altered mental status	152	559	27.2%
Seizure	70	625	11.2%
Pituitary dysfunction	21	205	10.2%
Incidental diagnosis	48	531	9.1%
**Tumor Radiographic Characteristics**			
Mean volume (cm^3^)	14.8	710	-
Mean diameter (mm)	38.6	380	-
Optic canal involvement	415	897	46.3%
Vascular involvement	119	405	29.4%
Peritumoral edema	185	559	33.1%

**Table 6:** Outcomes of transcranial surgery for anterior midline skull base meningiomas by tumor location.

Variable	OG			TS + PS		
	Event (*n*)	Total (*N*)	(%)	Event (*n*)	Total (*N*)	(%)
**Extent of Resection**						
GTR	329	358	91.9%	1024	1176	87.1%
STR	29	358	8.1%	150	1176	12.8%
**Visual Outcomes**						
**Preoperative**						
Impaired	59	358	16.5%	940	1174	80%
Postoperative						
Improved	27	358	7.5%	589	966	61%
Stable (impaired)	27	358	7.5%	278	966	28.8%
Worsened	11	358	3.1%	99	966	10.2%
**Olfactory Outcomes**						
**Preoperative**						
Normosmia	181	340	53.2%	465	1016	45.8%
Hyposmia	88	340	25.9%	5	1016	0.5%
Anosmia	71	340	20.9%	18	1016	1.8%
**Postoperative**						
Normosmia	59	340	17.4%	694	1016	68.3%
Hyposmia	40	340	11.8%	67	1016	6.6%
Anosmia	120	340	35.3%	36	1016	3.5%
**Postoperative Complications**						
CSF leakage	38	358	10.6%	13	1178	1.1%
Meningitis/infection	12	358	3.4%	33	1178	2.8%
Endocrine dysfunction	0	358	0%	71	1178	6%
Mortality						
Surgical (perioperative)	5	358	1.4%	1	1178	0.1%
Non-surgical	1	358	0.3%	8	1178	0.7%
Recurrence	5	358	1.4%	53	1029	5.2%
Follow-up duration (months; mean (range))	33.84 (14.5–62)	358		40.6 (12–62)	1029	

CSF, cerebrospinal fluid; GTR, gross total resection; OG, olfactory groove; PS, planum sphenoidale; STR, subtotal resection; TS, tuberculum sellae; TS + PS, tuberculum sellae/planum sphenoidale.

## Data Availability

The data presented in this study are available on request from the corresponding author.

## Supplementary Materials

The following supporting information can be downloaded at: https://www.mdpi.com/article/10.3390/jcm15124676/s1; Table S1: Quality Assessment Tool for Observational Cohort and Cross-Sectional Studies (NIH); Table S2: JBI Critical Appraisal Checklist for Case Reports; Supplementary Data S1: PRISMA 2020 Checklist, Supplementary Data S2: Detailed search strategy used for all databases.

## Abbreviations

The following abbreviations are used in this manuscript:

ACA	Anterior cerebral artery
CSF	Cerebrospinal fluid
EEA	Endoscopic endonasal approach
GTR	Gross total resection
ICA	Internal carotid artery
JBI	Joanna Briggs Institute
NIH	National Institutes of Health
NR	Not reported
NSF	Nasoseptal flap
OGM	Olfactory groove meningioma
PSM	Planum sphenoidale meningioma
PRISMA	Preferred Reporting Items for Systematic Reviews and Meta-Analyses
PROSPERO	International Prospective Registry of Systematic Reviews
STR	Subtotal resection
TCA	Transcranial approach
TSM	Tuberculum sellae meningioma
TS/PS	Tuberculum sellae/planum sphenoidale
