# Supplementary material for: Anterior Midline Skull Base Meningiomas: A Systematic Review of Resection Rates, Functional Outcomes, and Perioperative Complications Following Contemporary Endoscopic Endonasal Versus Transcranial Approaches
Source: J Clin Med. 2026 Jun 16;15(12):4676. doi: 10.3390/jcm15124676 (PMC13301445; doi:10.3390/jcm15124676)
Supplement: Supplementary file 1 [file jcm-15-04676-s001.zip › jcm-4310843-Supplementary Data S1-PRISMA 2020 Checklist.pdf]

PRISMA 2020 Checklist

Section and Topic	Item #	Checklist item	Location where item is reported
**TITLE**			
Title	1	Identify the report as a systematic review.	Title page - manuscript title explicitly states "Systematic Review"
**ABSTRACT**			
Abstract	2	See the PRISMA 2020 for Abstracts checklist.	Abstract (Title page)-structured abstract with Objectives, Methods, Results, Conclusions
**INTRODUCTION**			
Rationale	3	Describe the rationale for the review in the context of existing knowledge.	Introduction, paragraphs 1–4 — rationale described in context of NSF adoption and evolving EEA indications
Objectives	4	Provide an explicit statement of the objective(s) or question(s) the review addresses.	Introduction, paragraph 5 — "our objective was to evaluate how the adoption of the NSF has reshaped these operative and functional outcomes"
**METHODS**			
Eligibility criteria	5	Specify the inclusion and exclusion criteria for the review and how studies were grouped for the syntheses.	Section 2, Eligibility Criteria — inclusion criteria (1–5) and exclusion criteria (1–3) listed explicitly; studies grouped by EEA vs TCA and by tumor subtype (OGM, TS/PS)
Information sources	6	Specify all databases, registers, websites, organisations, reference lists and other sources searched or consulted to identify studies. Specify the date when each source was last searched or consulted.	Section 2, Study Identification — PubMed, Scopus,

PRISMA 2020 Checklist

Section and Topic	Item #	Checklist item	Location where item is reported
			Embase searched; studies from 2010 onward; reference lists of included studies examined. Note: exact search date not specified — consider adding.
Search strategy	7	Present the full search strategies for all databases, registers and websites, including any filters and limits used.	Section 2, Study Identification — full Boolean search string provided; filters: publication year ≥2010, English language
Selection process	8	Specify the methods used to decide whether a study met the inclusion criteria of the review, including how many reviewers screened each record and each report retrieved, whether they worked independently, and if applicable, details of automation tools used in the process.	Section 2, Study Selection Process — 2 independent reviewers (U.S., I.U.) screened titles/abstracts; 3 reviewers (U.S., I.U., Y.S.) assessed full texts; disagreements resolved by consensus
Data collection process	9	Specify the methods used to collect data from reports, including how many reviewers collected data from each report, whether they worked independently, any processes for obtaining or confirming data from study investigators, and if applicable, details of automation tools used in the process.	Section 2, Data Extraction — 2 independent reviewers; standardized protocol; discrepancies resolved by discussion and consensus
Data items	10a	List and define all outcomes for which data were sought. Specify whether all results that were compatible with each outcome domain in each study were sought (e.g. for all measures, time points, analyses), and if not, the methods used to decide which results to collect.	Section 2, Data Extraction — outcomes listed: extent of resection (GTR/STR), visual outcomes, olfactory outcomes, complications (CSF leak, meningitis,

Section and Topic	Item #	Checklist item	Location where item is reported
			endocrine dysfunction, mortality), recurrence
	10b	List and define all other variables for which data were sought (e.g. participant and intervention characteristics, funding sources). Describe any assumptions made about any missing or unclear information.	Section 2, Data Extraction — variables: study authors, year, sample size, patient demographics, presenting symptoms, tumor radiographic characteristics; missing/unclear data noted as NR or excluded from pooled denominators
Study risk of bias assessment	11	Specify the methods used to assess risk of bias in the included studies, including details of the tool(s) used, how many reviewers assessed each study and whether they worked independently, and if applicable, details of automation tools used in the process.	Section 2, Risk of Bias Assessment — NIH Quality Assessment Tool (cohort studies) and JBI critical appraisal tool (case series); 2 reviewers (O.A., I.U.) independently; disagreements resolved by third author
Effect measures	12	Specify for each outcome the effect measure(s) (e.g. risk ratio, mean difference) used in the synthesis or presentation of results.	Section 2, Data Analysis — descriptive review; outcomes reported as absolute event counts and percentages; continuous variables as pooled means ± SD; no formal effect measures (no meta-analysis)
Synthesis	13a	Describe the processes used to decide which studies were eligible for each synthesis (e.g. tabulating the study intervention	Section 2, Data

Section and Topic	Item #	Checklist item	Location where item is reported
methods		characteristics and comparing against the planned groups for each synthesis (item #5)).	Analysis — studies grouped by surgical approach (EEA vs TCA) and tumor subtype (OGM vs TS/PS); comparative studies included if approach-specific outcomes were extractable
	13b	Describe any methods required to prepare the data for presentation or synthesis, such as handling of missing summary statistics, or data conversions.	Section 2, Data Analysis — continuous variables converted using Hozo et al. method when reported as median/range; missing data excluded from denominators
	13c	Describe any methods used to tabulate or visually display results of individual studies and syntheses.	Section 2, Data Analysis — results tabulated in Tables 1–6; flow diagram in Figure 1; narrative synthesis by tumor subtype
	13d	Describe any methods used to synthesize results and provide a rationale for the choice(s). If meta-analysis was performed, describe the model(s), method(s) to identify the presence and extent of statistical heterogeneity, and software package(s) used.	Section 2, Data Analysis — descriptive systematic review; no meta-analysis performed due to study heterogeneity; data aggregated using R statistical software
	13e	Describe any methods used to explore possible causes of heterogeneity among study results (e.g. subgroup analysis, meta-regression).	Section 2, Data Analysis — no formal heterogeneity analysis; subgroup descriptions for OGM, TSM, PSM

Section and Topic	Item #	Checklist item	Location where item is reported
			provided narratively
	13f	Describe any sensitivity analyses conducted to assess robustness of the synthesized results.	Not performed — not applicable to this descriptive systematic review (noted as a limitation)
Reporting bias assessment	14	Describe any methods used to assess risk of bias due to missing results in a synthesis (arising from reporting biases).	Not formally assessed; Section 5 (Limitations) acknowledges selection and reporting bias as inherent limitations of included retrospective cohort studies
Certainty assessment	15	Describe any methods used to assess certainty (or confidence) in the body of evidence for an outcome.	Not formally performed (e.g., GRADE not used); risk of bias assessed using NIH and JBI tools (Section 2, Risk of Bias Assessment)
**RESULTS**			
Study selection	16a	Describe the results of the search and selection process, from the number of records identified in the search to the number of studies included in the review, ideally using a flow diagram.	Section 3.1, Study Selection — 35 studies included; PRISMA flow diagram in Figure 1
	16b	Cite studies that might appear to meet the inclusion criteria, but which were excluded, and explain why they were excluded.	Section 3.1 — exclusion criteria applied (keyhole/combined approaches, no primary data, recurrent tumors only); specific excluded studies not cited individually — consider adding if required by journal

PRISMA

PRISMA 2020 Checklist

Section and Topic	Item #	Checklist item	Location where item is reported
Study characteristics	17	Cite each included study and present its characteristics.	Tables 1 and 4 — all 35 included studies cited with year, country, study design, approach, aim, and conclusion
Risk of bias in studies	18	Present assessments of risk of bias for each included study.	Section 3.4 — quality ratings summarized; detailed assessments in Supplementary Tables S1–S2
Results of individual studies	19	For all outcomes, present, for each study: (a) summary statistics for each group (where appropriate) and (b) an effect estimate and its precision (e.g. confidence/credible interval), ideally using structured tables or plots.	Sections 3.2–3.3 and Tables 2–6 — pooled outcomes presented by approach and tumor subtype; individual study data in Tables 1 and 4
Results of syntheses	20a	For each synthesis, briefly summarise the characteristics and risk of bias among contributing studies.	Sections 3.2–3.3 — study characteristics summarized by approach group; risk of bias summary in Section 3.4
	20b	Present results of all statistical syntheses conducted. If meta-analysis was done, present for each the summary estimate and its precision (e.g. confidence/credible interval) and measures of statistical heterogeneity. If comparing groups, describe the direction of the effect.	Tables 3 and 6 — pooled outcomes by tumor subtype for EEA and TCA; no meta-analysis performed
	20c	Present results of all investigations of possible causes of heterogeneity among study results.	Not performed — heterogeneity addressed narratively in Sections 4 and 5
	20d	Present results of all sensitivity analyses conducted to assess the robustness of the synthesized results.	Not performed — not applicable to this descriptive

Section and Topic	Item #	Checklist item	Location where item is reported
			systematic review
Reporting biases	21	Present assessments of risk of bias due to missing results (arising from reporting biases) for each synthesis assessed.	Not formally assessed; acknowledged in Section 5 (Limitations)
Certainty of evidence	22	Present assessments of certainty (or confidence) in the body of evidence for each outcome assessed.	Not formally assessed using GRADE; study quality reported using NIH and JBI tools (Section 3.4 and Supplementary Tables S1–S2)
**DISCUSSION**			
Discussion	23a	Provide a general interpretation of the results in the context of other evidence.	Section 4 (Discussion) — results interpreted in context of prior literature including Komotar et al. 2012 and contemporary series
	23b	Discuss any limitations of the evidence included in the review.	Section 5 (Limitations) — limitations of included studies discussed: retrospective design, selection bias, reporting bias, heterogeneous outcome measures
	23c	Discuss any limitations of the review processes used.	Section 5 (Limitations) — limitations of review process discussed: heterogeneity precluding meta-analysis, inconsistent tumor characteristic reporting, variable follow-up duration

PRISMA

PRISMA 2020 Checklist

Section and Topic	Item #	Checklist item	Location where item is reported
	23d	Discuss implications of the results for practice, policy, and future research.	Sections 4 and 6 — implications for surgical decision-making discussed; future studies with dedicated PSM cohorts recommended
**OTHER INFORMATION**			
Registration and protocol	24a	Provide registration information for the review, including register name and registration number, or state that the review was not registered.	Section 2 — PROSPERO registration: CRD420251147118
	24b	Indicate where the review protocol can be accessed, or state that a protocol was not prepared.	Not explicitly stated — consider adding statement about protocol availability
	24c	Describe and explain any amendments to information provided at registration or in the protocol.	Not reported — no amendments described
Support	25	Describe sources of financial or non-financial support for the review, and the role of the funders or sponsors in the review.	Funding section — "This research received no external funding"
Competing interests	26	Declare any competing interests of review authors.	Conflicts of Interest section — "The authors declare no conflicts of interest"
Availability of data, code and other materials	27	Report which of the following are publicly available and where they can be found: template data collection forms; data extracted from included studies; data used for all analyses; analytic code; any other materials used in the review.	Data Availability Statement — "data presented in this study are available on request from the corresponding author"; Supplementary Materials reference Tables S1–S2

From: Page MJ, McKenzie JE, Bossuyt PM, Boutron I, Hoffmann TC, Mulrow CD, et al. The PRISMA 2020 statement: an updated guideline for reporting systematic reviews. BMJ 2021;372:n71. doi: 10.1136/bmj.n71. This work is licensed under CC BY 4.0. To view a copy of this license, visit https://creativecommons.org/licenses/by/4.0/
