# Supplementary material for: Anterior Midline Skull Base Meningiomas: A Systematic Review of Resection Rates, Functional Outcomes, and Perioperative Complications Following Contemporary Endoscopic Endonasal Versus Transcranial Approaches
Source: J Clin Med. 2026 Jun 16;15(12):4676. doi: 10.3390/jcm15124676 (PMC13301445; doi:10.3390/jcm15124676)
Supplement: Supplementary file 1 [file jcm-15-04676-s001.zip › jcm-4310843-Supplementary Data S2.pdf]

Article title: Anterior Midline Skull Base Meningiomas: A Systematic Review of Resection Rates, Functional Outcomes, and Perioperative Complications Following Contemporary Endoscopic Endonasal Versus Transcranial Approaches

Journal: Journal of Clinical Medicine

Authors: Umid Sulaimanov, Irem Uslu, Omar Alomari, Yerkebulan Serikkanov, Mariagrazia Nizzola, Darius Ansari, Oyku Ozturk, Nafiye Sanlier, Ahmed Rasim Bayramoglu, Eray Tekirdas, Abdullah Keles, Ufuk Erginoglu and Mustafa K. Baskaya

Corresponding author: Mustafa K. Baskaya

Supplementary Data S2: Detailed search strategy used for all databases

#1	("Meningiomas"[Mesh] OR "Tuberculum Sellae Meningioma" OR "Olfactory Groove Meningioma" OR "Planum Sphenoidale Meningioma" OR "Anterior Skull Base Meningioma"
#2	("Craniotomy"[Mesh] OR "craniotomy" OR "transcranial" OR "open approach" OR "transcranial resection"<br>OR "subfrontal approach" OR "frontotemporal approach" OR "bifrontal approach" OR "pterional approach"<br>OR "lateral supraorbital approach" OR "lateral suboccipital approach"<br>OR "Subfrontal Approach"[Mesh] OR "Frontotemporal Approach"[Mesh] OR "Bifrontal Approach"[Mesh]<br>OR "Pterional Approach"[Mesh] OR "Lateral Supraorbital Approach"[Mesh] OR "Lateral Suboccipital Approach"[Mesh])
#3	"Surgical Procedures, Operative"[Mesh] OR "resection" OR "removal")
#4	(humans[MeSH Terms])
#5	#1 AND #2 AND #3 AND #4

#1	"Meningiomas"[Mesh] OR "Tuberculum Sellae Meningioma" OR "Olfactory Groove Meningioma" OR "Planum Sphenoidale Meningioma" OR "Anterior Skull Base Meningioma")
#2	("Endoscopy"[Mesh] OR "endoscopic" OR "endonasal" OR "endoscopic endonasal approach")

#3	("Surgical Procedures, Operative"[Mesh] OR "resection" OR "removal")
#4	(humans[MeSH Terms])
	#1 AND #2 AND #3 AND #4
